# Supplementary material for: Validation of carbon isotopologue distribution measurements by GC-MS and application to 13C-metabolic flux analysis of the tricarboxylic acid cycle in Brassica napus leaves
Source: Front Plant Sci. 2023 Jan 10;13:885051. doi: 10.3389/fpls.2022.885051 (PMC9871494; doi:10.3389/fpls.2022.885051)
Supplement: Supplementary file 2 [file DataSheet_2.zip › Supplementary_Figures.docx]

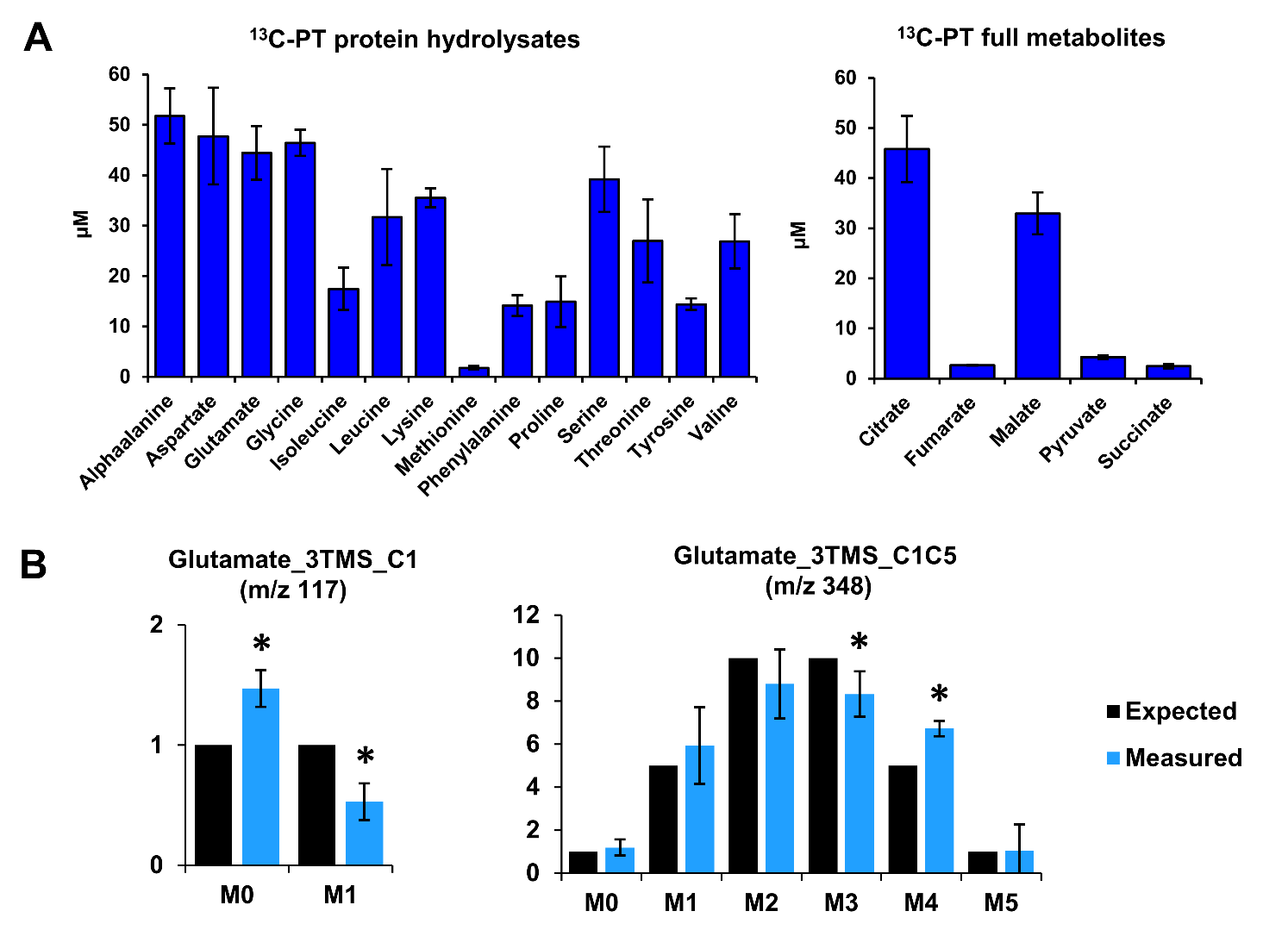


**Supplementary Figure 1.** **A**, Absolute quantification of organic and amino acids in 13C-PT samples using GC-FID and HPLC-UV methods. **B**, Relative CID measured in 13C-PT protein hydrolysates for Glutamate_3TMS_C1 and Glutamate_3TMS_C1C5 fragments. The results are presented as the mean ± SD of four independent biological replicates. Statistical differences between predicted and measured CID for each isotopologue of each fragment are denoted with asterisks (*) and were established by considering the 95% confidence intervals of the measured CID. GC-MS fragments are denoted according to the considered metabolite, its MEOX/TMS-derivatives analyzed and the metabolite carbon backbone (for example, Glutamate_3TMS_C2C5 means that the fragment comes from Glutamate(3TMS) and contains the carbon C2-C3-C4-C5 (**Table 1**)).


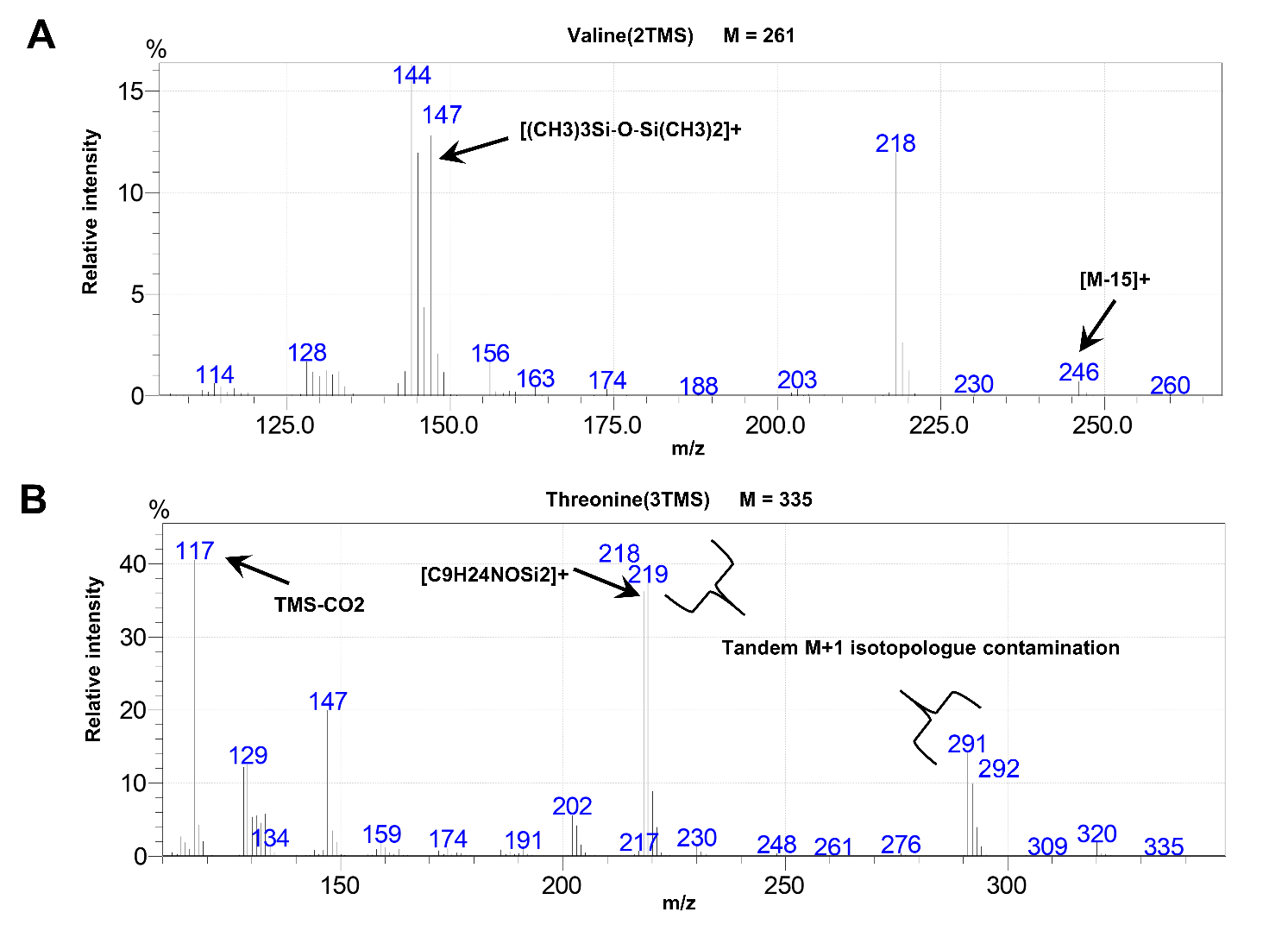


**Supplementary Figure 2.** Zoom on the fragmentation spectra and contaminations of **A**, Valine_2TMS and **B**, Threonine_3TMS with unlabeled Arabidopsis seedlings.


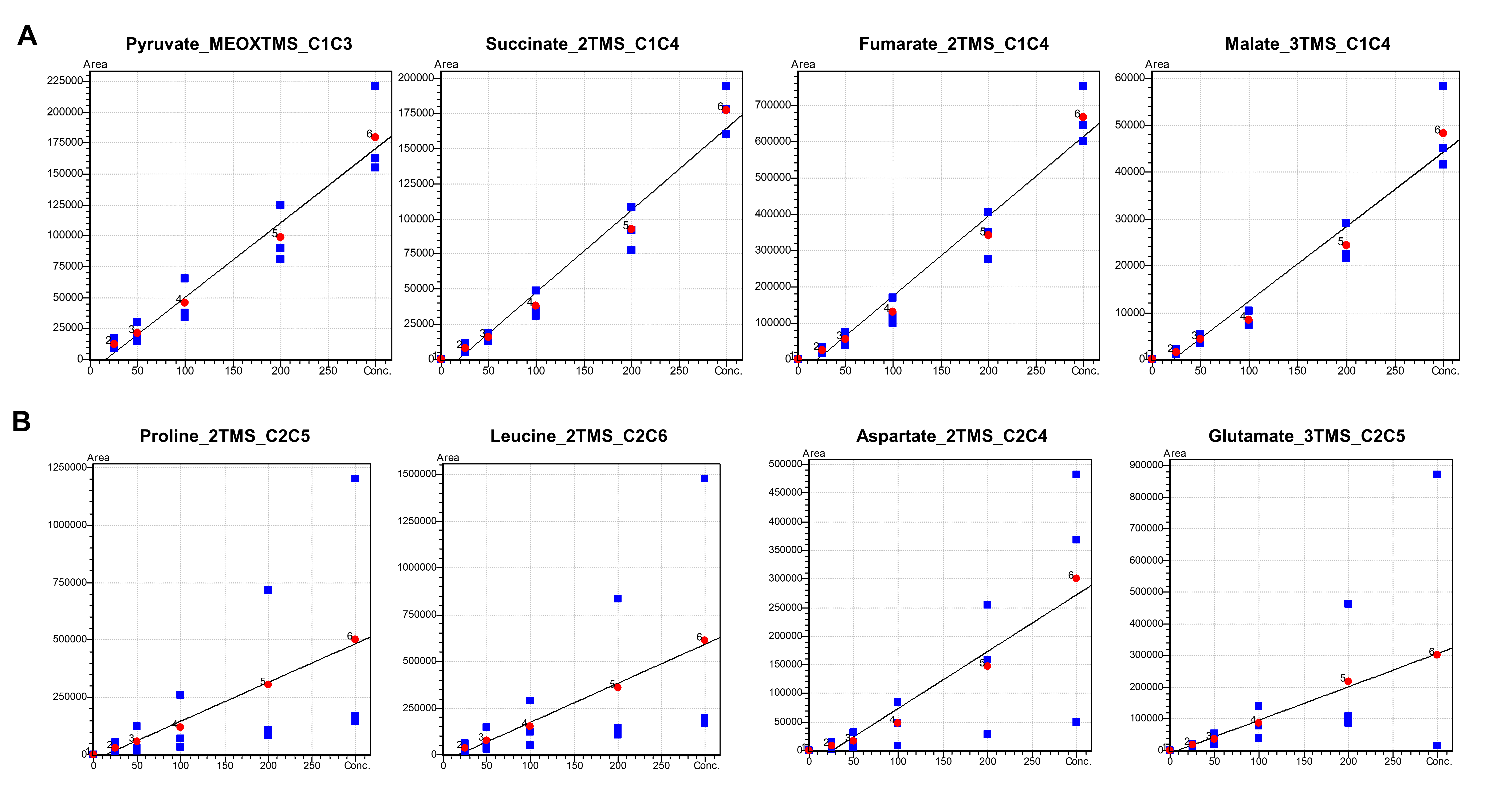


**Supplementary Figure 3.** GC-MS calibration curves for some **A**, organic acids and **B**, amino acids. Calibration was tested with four independent preparations of the following concentrations: 0, 25, 50, 100, 200, 300 µM. All the samples received the same treatment, including the same resting time before injection. They were injected in the same run to account for time-dependent modulation of the coefficient response. The calibration curves are based on M0 isotopologues, after correcting for naturally occurring isotopes. Blue dots are for original values and red dots are for the mean value.
